# c-Rel Is Required for IL-33-Dependent Activation of ILC2s

**DOI:** 10.3389/fimmu.2021.667922

**Published:** 2021-06-14

**Authors:** Aidil Zaini, Thomas S. Fulford, Raelene J. Grumont, Jessica Runting, Grace Rodrigues, Judy Ng, Steve Gerondakis, Colby Zaph, Sebastian Scheer

**Affiliations:** ^1^ Infection and Immunity Program, Monash Biomedicine Discovery Institute, Clayton, VIC, Australia; ^2^ Department of Biochemistry and Molecular Biology, Monash University, Clayton, VIC, Australia; ^3^ Department of Microbiology and Immunology, University of Melbourne, The Peter Doherty Institute for Infection and Immunity, Melbourne, VIC, Australia

**Keywords:** ILC2, c-Rel, allergic lung inflammation, IL-33, papain

## Abstract

Group 2 innate lymphoid cells (ILC2s) are emerging as important cellular regulators of homeostatic and disease-associated immune processes. The cytokine interleukin-33 (IL-33) promotes ILC2-dependent inflammation and immunity, with IL-33 having been shown to activate NF-κB in a wide variety of cell types. However, it is currently unclear which NF-κB members play an important role in IL-33-dependent ILC2 biology. Here, we identify the NF-κB family member c-Rel as a critical component of the IL-33-dependent activation of ILC2s. Although c-Rel is dispensable for ILC2 development, it is critical for ILC2 function in the lung, with c-Rel-deficient (*c-Rel^–/–^*) mice present a significantly reduced response to papain- and IL-33-induced lung inflammation. We also show that the absence of c-Rel reduces the IL-33-dependent expansion of ILC2 precursors and lower levels of IL-5 and IL-13 cytokine production by mature ILC2s in the lung. Together, these results identify the IL-33-c-Rel axis as a central control point of ILC2 activation and function.

## Introduction

Innate lymphoid cells (ILCs) are important regulators of innate and adaptive immune responses, including inflammatory and allergic responses, as well as in homeostatic processes at barrier tissues (1). ILC subsets are distinguished by distinct developmental pathways, transcription factor expression and production of effector cytokines. Group 1 ILCs (ILC1s) produce IFN-γ and express T-BET, group 2 ILCs (ILC2s) express IL-5, IL-13 and GATA-3, and group 3 ILCs (ILC3s) produce IL-17 and IL-22, and express RORγt (1). Of all ILC subsets, ILC2s are the most important subtype for regulating type 2 immune responses and thus serve key roles in mucosal homeostasis, allergy and anti-helminth immunity. ILC2s primarily reside at mucosal barrier surfaces, highlighting their importance as a first line of defense against invading pathogens. Upon epithelial cell damage, the alarmin IL-33 is released by epithelial cells and binds to a heterodimeric receptor expressed on immune cells, including ILC2s that results in cellular activation (2). Furthermore, IL-33 has been shown to regulate ILC2 mobilization from the bone marrow to the lungs (3). In addition to IL-33, the epithelial cell-derived cytokines IL-25 and TSLP also have important roles in ILC2 activation and function in response to helminth infections (4), as well as type 2 inflammatory responses such as allergy and asthma (5–7). However, the precise molecular mechanisms of IL-33-induced ILC2 activation remain unclear.

NF-κB comprises a group of transcription factors that play diverse and often critical roles in innate and adaptive immune responses. In mammals, there are five NF-κB family members, namely RelA, RelB, c-Rel, NF-κB1 (p50) and NF-κB2 (p100), all of which share a Rel homology domain (8). NF-κB proteins exist as either homodimers or heterodimers that normally reside within the cytoplasm in an inactive state. These proteins are rapidly activated in response to diverse upstream signals that typically engage one of two activation pathways: the canonical and non-canonical pathways (9). The canonical pathway activates NF-κB *via* the Iκκβ-dependent phosphorylation and subsequent degradation of the cytoplasmic inhibitory protein IκBα, thereby to allow RelA, c-Rel and p50 homo- and heterodimers to translocate to the nucleus and regulate gene expression. In contrast, the non-canonical pathway relies upon IKKα phosphorylation-dependent activation of the NF-κB family members p100 and RelB, allowing translocation of p52/RelB heterodimers to the nucleus (10). Although IL-33 has been shown to activate NF-κB in a wide variety of immune cells (2), the precise molecular mechanisms involved are unknown, as are the composition of activated dimers in different cell types, including ILCs.

ST2 is one of the subunits of the IL-33 receptor that signals *via* MYD88-dependent NF-κB activation to modulate distinct gene expression programs (2, 11, 12). Canonical NF-κB signaling has been shown to regulate GATA3 expression in T helper 2 (Th2) cells (13), and T regulatory (Treg) cells (14, 15). Furthermore, IL-33-ST2 signaling is associated with the development and function of Th9 cells (16), dendritic cells (17, 18), and macrophages (19). IL-33-ST2 signaling is also critical for ILC2 function, although whether canonical NF-κB is required remains unclear (20, 21). Recently, non-canonical NF-κB signaling has been shown to be required for IL-33-dependent ILC2s in adipose tissue following death receptor 3 engagement (22), as well as in pulmonary ILC2s upon tumor necrosis factor receptor 2 binding (23). However, adiponectin treatment used to activate the energy sensor AMP-activated protein kinase inhibits the phosphorylation of IKKα/β and IκBα in IL-33-activated adipose-resident ILC2s and impairs IL-13 production (24), suggesting that canonical NF-κB signaling may also play an important role in ILC2 activation and function.

In this present study, we identify a role for the canonical NF-κB family member c-Rel in ILC2 biology under both homeostatic and inflammatory conditions. Our results suggest that while c-Rel is dispensable for ILC2 development in the bone marrow (BM), it is required for peripheral IL-33-dependent ILC2 activation and the development of allergic lung inflammation. These findings point to c-Rel as a potential therapeutic target for treating ILC2-dependent lung inflammation.

## Materials and Methods

### Mice

C57BL/6J mice (wild-type), c-Rel-deficient mice on C57BL/6J background (c-Rel^-/-^), NF-κB1 (p105/p50)-deficient mice on C57BL/6J background (NF-κB1^-/-^) (25) were bred and kept at Monash University. Animals used in this study were 7 to 10 weeks old, with mice maintained under specific-pathogen-free conditions (SPF) conditions. All studies were performed at Monash Biomedicine Discovery Institute (BDI), Monash University in accordance with Monash Animal Ethics Committee (AEC) and Australian National Health and Medical Research Council (NHMRC) guidelines for animal experimentation.

### Electrophoretic Mobility Shift Assay (EMSA)

Nuclear extract preparation was performed as previously described (26). 1-2 μg of nuclear extracts prepared from IL-33-restimulated (6 h) BM-derived ILC2 precursors (ILC2Ps) were incubated with a κB3-specific ^32^P-dATP end-labelled probe, as previously described (27). For supershift analysis, antibodies against p50, c-Rel and RelA were incubated with nuclear extracts on ice for 30 min before adding the radiolabeled probe (27). The samples were incubated for 20 min at room temperature, then 2 μl of gel loading dye Ficoll was added, and the samples fractionated on 5% non-denaturing polyacrylamide gels. Gels were dried and exposed to autoradiography.

### Preparation of Single Cell Suspension

BM cells were isolated from femur and tibia by flushing the BM using a 25G needle and passing the cells through a 70-μm strainer to form a single cell suspension. For some experiments, lungs were cut in small pieces and digested in 400 U/ml collagenase IV (Sigma Aldrich), followed by incubation at 37°C for 45 min in complete RPMI media (Life Technologies). The digested lungs were then passed through a 70-μm strainer. Red blood cells (RBCs) in organ cell suspensions were lysed in 1 ml RBC lysis buffer (eBioscience) for 1 min. After two washes in FACS buffer, the samples were resuspended in a 30% Percoll separation solution and centrifuged at 400 x *g* to enrich the leukocytes, followed by staining for flow cytometry analysis.

### Flow Cytometry

Cells were first blocked with purified rat anti-mouse CD16/CD32 (2 μg/ml) (eBioscience) and rat serum (20 μg/ml) (Stem Cell). Cells were then stained with specific antibodies of interest in the FACS buffer (**Supplementary Table 1**) (2% FCS, 1 mM EDTA, and 0.05% azide in PBS). For BM-derived ILC2Ps FACS sorted for culture, the cells were stained in ILC media. Viable cells were identified using the viability dye 7-AAD (eBioscience). The samples were either resuspended in the FACS buffer for acquisition, or fixed overnight at 4°C for intracellular staining the next day using a FOXP3 kit (Tonbo Biosciences). The staining was performed according to the manufacturer’s instructions.

### Cell Culture

Bone marrow-derived ILC2Ps or lung ILC2s were sorted by flow cytometry following standard protocols. In short, 5,000 ILC2s were cultured in round bottom plates containing ILC media in the presence of IL-2 (50 ng/ml), IL-7 (10 ng/ml) and IL-25 (100 ng/ml) at d0. Cells were split 1/2 with fresh ILC media plus IL-2 (50 ng/ml), IL-7 (10 ng/ml) and IL-25 (100 ng/ml) at d3 and every second day thereafter over a period of 14 days. On d15, 200,000 cells were plated per well in ILC media in the presence of IL-2 (50 ng/ml) and IL-7 (10 ng/ml) for 24 h. On the following day, the cells were restimulated with IL-33 (10 ng/ml) for 6 h. For some experiments, 5,000 lung-derived ILC2s were cultured overnight in ILC media plus IL-2 (50 ng/ml), IL-7 (10 ng/ml), IL-25 (100 ng/ml) and IL-33 (10 ng/ml) with pentoxifylline (500 ng/ml).

### Lung Inflammation Model of Asthma

Mice were intranasally instilled with 10 μg of papain (Sigma-Aldrich) in 40 μl of PBS under isoflurane anesthesia daily for 3 days. For rIL-33-induced lung inflammation, mice were intranasally injected with 500 ng IL-33 (eBiosciences) daily for 3 days, as previously described (21). 24 h after the last treatment, mice were sacrificed, and their bronchoalveolar lavage (BAL) fluid and lung tissues were collected for flow cytometry analysis and RNA extraction. The left lobe of the lung was obtained and fixed in 10% Formalin solution. The lung tissue was embedded into paraffin blocks and were stained with periodic acid-Schiff (PAS) stain, as previously described (28).

### qPCR

RNA from homogenized tissue was isolated using phenol-chloroform extraction as per standard protocol. RNA from cultured ILC2s was extracted using a NucleoSpin RNA Kit according to manufacturer’s instructions (MACHEREY-NAGEL). The concentration and purity of extracted RNA was measured using a Spectrophotometer NanoDrop 1000 (Thermo Fisher Scientific). 1 μg of RNA was used for cDNA generation using a cDNA conversion kit (Thermo Fisher Scientific). qPCR was performed using a SYBR green chemistry (Qiagen) on a qPCR system (Rotor-Gene Q Qiagen) using specific primers (**Supplementary Table 2**). Samples were standardized using *Actb*.

### ELISA

ELISA plates were coated with primary antibodies at 1 μg/ml in PBS at 4°C overnight. The plates were washed 4 times using a washing buffer (PBS containing 0.05% Tween 20) (Sigma Aldrich) with 1 min rests between washes. Subsequently, the plates were blocked for 1 h at room temperature with 200 μl PBS containing 10% newborn calf serum (Bovogen). The samples were loaded, followed by a two-hour incubation at room temperature. The plates were washed 5 times, and the secondary antibodies (biotinylated) were loaded (0.5 μg/ml), and incubated for 1 h at room temperature, followed by 8 final washes with washing buffer. TMB substrate (Invitrogen) was added, and the reaction was stopped with HCL. The samples were read at 450 nm using a microplate Epoch spectrophotometer (BioTek) and analyzed using Gen5 2.0 (data analysis software).

### Statistics

All statistical calculations were performed with GraphPad Prism 9 software (GraphPad Software, La Jolla, CA, USA). All graphs represent mean ± SEM. Statistical significance was determined by 2-tailed Student’s t test or one-way-ANOVA. Results were considered statistically significant with p ≤ 0.05.

## Results

### Canonical NF-κB Activity in ILC2s

**Figure 1:**
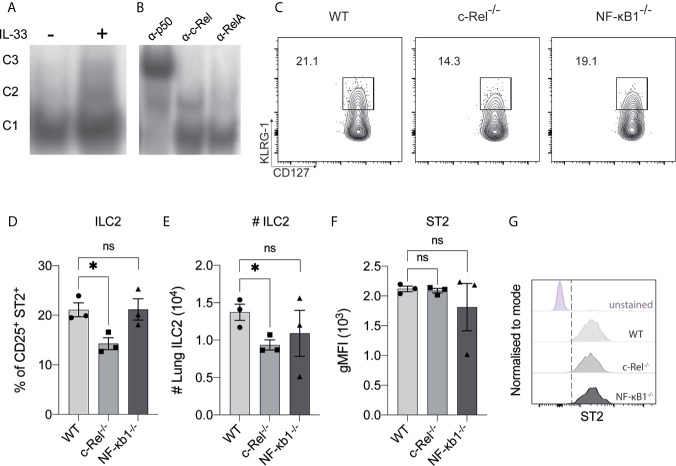
The presence of canonical NF-κB activity in ILC2s. **(A)** Electrophoretic mobility shift assay (EMSA) analysis in IL-33-stimulated BM-derived ILC2Ps. **(B)** The specific members of NF-κβ were detected *via* antibody supershift analysis by using specific antibodies against p50, cRel and RelA. C1 is composed of p50, C2 is composed of RelA and C3 is composed of c-Rel protein. **(C)** Representative flow cytometry plot of CD127^+^ KLRG-1^+^ lung ILC2s (gated on viable CD45^+^ Lin^-^ CD90^+^ CD25^+^ ST2^+^ cells) from WT, c-Rel^-/-^ and NF-κB^-/-^ naive mice. **(D)** Quantification of lung ILC2 among parent CD25^+^ ST2^+^ cells. **(E)** Absolute numbers of lung ILC2s per animal. **(F)** Quantification of ST2 in ILC2s, expressed as geometric mean fluorescence intensity (gMFI). **(G)** Representative histogram plots of ST2 expression in ILC2s. All plots and graphs are representative of at least two independent experiments with at least three mice per group. Error bars represent ± SEM. *p ≤ 0.05. ns, non-significant.

To begin investigating NF-κB involvement in the molecular requirements for IL-33-dependent activation of ILC2s, we examined IL-33-dependent NF-κB activation in ILC2s by electrophoretic mobility shift assay (EMSA). We used flow cytometry to isolate Lin^-^ CD45^+^ c-KIT^-^ Sca-1^+^ CD127^+^ CD25^+^ α^4^β^7+^ ILC2Ps from WT mice and expanded them *in vitro* with IL-2, IL-7, IL-25 and IL-33 for 2 weeks. Expanded ILC2s were rested for 48 h in IL-2 and IL-7, and subsequently restimulated with IL-33 for 6 h. IL-33 restimulation led to increased nuclear translocation and DNA binding of three distinct NF-κB complexes in ILC2s that are denoted as C1 (lower band), C2 (middle band) and C3 (upper band) (**Figure 1A**). Antibody super-shifts revealed that the three complexes were comprised of the canonical NF-κB family members p50 (i.e. NF-κB1 component) (C1), RelA (C2) and c-Rel (C3) (**Figure 1B**). Thus, ILC2s respond to IL-33 stimulation by activating the canonical NF-κB pathway.

We next examined the frequencies of ILC2s in the lungs of c-Rel-deficient (c-Rel^-/-^) and p105/p50-deficient (NF-κB1^-/-^) mice. We did not examine the role of RelA as RelA-deficient mice are perinatal lethal (29). We found the frequencies of lung ILC2s were subtly, but significantly reduced by the absence of c-Rel, while NF-κB1*^-/-^* mice had a non-significant reduction in ILC2 frequency and numbers (**Figures 1C–E**). We also found that the absence of c-Rel or NF-κB1 had no effect on the expression level of the IL-33 receptor component ST2 in ILC2s (**Figures 1F, G**). Thus, deficiency of the NF-κB family member c-Rel has a minimal yet significant impact on the homeostatic levels of ILC2s in the lungs.

### c-Rel Is Required for IL-33-Dependent ILC2 Expansion but Not ILC2 Development in the Bone Marrow

**Figure 2:**
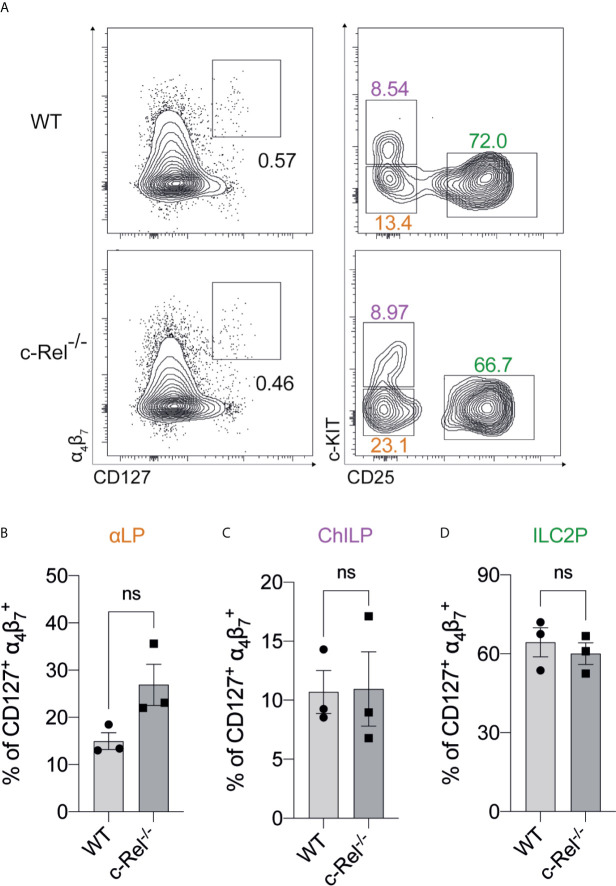
The development of BM-derived immature ILC2s are independent of c-Rel **(A)** Representative flow cytometry plot of α_4_β_7_^+^ CD127^+^ immature BM ILC2s (left) (gated on viable CD45^+^ Lin^-^ cells) and specific ILC2 progenitors (gated on parent α_4_β_7_^+^ CD127^+^ cells). α-lymphoid progenitors (αLPs) were defined as c-KIT^low^ CD25^-^, whereas common helper innate lymphoid progenitors (ChILPs) and ILC2 precursors (ILC2Ps) were c-KIT^high^ CD25^-^ and c-KIT^low^ CD25^+^, respectively. **(B–D)** Quantification of αLP, ChILP and ILC2P among α_4_β_7_^+^ CD127^+^ cells, respectively. All plots and graphs are representative of at least two independent experiments with at least three mice per group. Error bars represent ± SEM. ns, non-significant.

**Figure 3:**
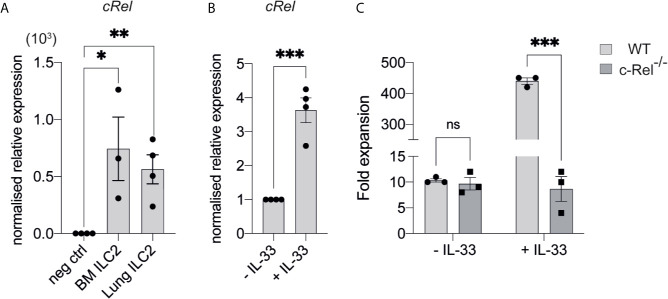
c-Rel is required for IL-33-dependent expansion of ILC2s. **(A)** qPCR analysis of *cRel* in *ex vivo* FACS-sorted BM-derived ILC2 and lung-derived ILC2 from naive WT mice (fold change over negative control). **(B)** qPCR analysis of *cRel* in BM-derived ILC2 cultures restimulated with IL-33 for 6 h. **(C)** Quantification of fold expansion derived from cell count on *ex vivo* BM-derived ILCP cultures. FACS-sorted BM ILC2Ps were cultured in ILC media in the presence of IL-2, IL-7 and IL-25 for 14 days. On d15, expanded cells were cultured in only IL-2 and IL-7 overnight. On d16, expanded cells were re-stimulated with or without IL-33 (control) for 6 h, followed by cell count. All plots and graphs are representative of at least two independent experiments with at least three mice per group. Error bars represent ± SEM. *p ≤ 0.05, **p ≤ 0.01, ***p ≤ 0.001. ns, non-significant.

We focused our investigation on c-Rel and started by examining the development of immature ILC2s (iILC2s) in the bone marrow of c-Rel-deficient mice during steady state hematopoiesis. Flow cytometric analysis revealed equivalent frequencies of Lin^-^ CD127^+^ α_4_β_7_^+^ CD25^+^ c-KIT^low^ ILC2Ps (30–32) in the BM of control and c-Rel^-/-^ mice (**Figures 2A, D**). Further, cells upstream of ILC progenitors, including common lymphoid progenitors (CLPs), α-lymphoid progenitors (αLPs) and common helper innate lymphoid progenitors (ChILPs), were also equally represented in the BM of control and c-Rel^-/-^ mice (**Figures 2A–C** and **Supplementary Figure 1A**). Because GATA3 is crucial for ILC2 development (33), we also assessed GATA3 expression in *ex vivo* BM ILC2s. Similar levels of GATA3 expression in BM ILC2s in both control and c-Rel^-/-^ mice coincided with normal development of BM ILC2 observed in c-Rel^-/-^ mice (**Supplementary Figures 1B, C**). This is also consistent with the phenotype observed in our ILC2 cultures whereby a majority of both control and c-Rel^-/-^ cells remained Lin^-^ and CD45^+^ (>70%) with equal representation of CD25 and Sca-1 expression (**Supplementary Figure 2A**). In steady state, *cRel* is expressed in both *ex vivo* WT BM and lung ILC2s in comparison to c-Rel-deficient control cells and the levels of *cRel* expression are comparable in these two ILC2 populations (**Figure 3A**). To assess the correlation between changes in *cRel* expression in response to IL-33 stimulation, we cultured WT BM-derived ILC2s for 6 h in the presence of IL-33. *cRel* expression was dramatically increased in the presence of extracellular IL-33 (**Figure 3B**). In accordance with increased *cRel* expression in ILC2s to IL-33 stimulation, we did find that c-Rel was critical for the IL-33-dependent expansion of ILC2Ps. We isolated ILC2Ps from BM of control and c-Rel^-/-^ mice and stimulated them *in vitro* with IL-2, IL-7 and IL-25. Under this condition, we observed equivalent expansion of control and c-Rel-deficient ILC2Ps (**Figure 3C**), although to a significantly lesser extent than when IL-33 was present. However, unlike WT ILC2Ps, c-Rel-deficient ILC2Ps failed to expand in response to IL-33. Thus, c-Rel is critically involved in the IL-33-responsiveness of ILC2Ps.

### c-Rel Regulates Papain-Induced Lung Inflammation

**Figure 4:**
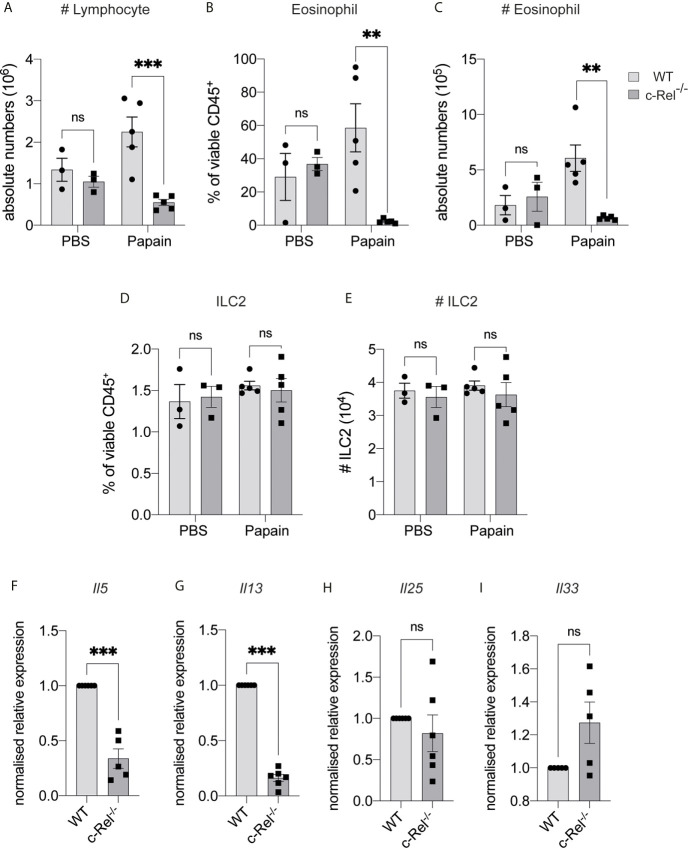
c-Rel deficiency reduces papain-induced lung inflammation. **(A)** Absolute numbers of CD45^+^ lymphocytes in the bronchoalveolar lavage (BAL) fluid following PBS (control) or papain challenge for three days. **(B)** Quantification of Siglec-F^+^ CD11c^-^ eosinophils in BAL among viable CD45^+^ cells. **(C)** Absolute numbers of BAL eosinophils. **(D)** Quantification of CD127^+^ KLRG-1^+^ lung ILC2s (gated on Lin^-^ CD90^+^ CD25^+^ ST2^+^) among viable CD45^+^ cells. **(E)** Absolute numbers of lung ILC2s per animal. **(F–I)** qPCR analysis of the indicated genes from lung tissue following papain challenge. All graphs are representative of at least two independent experiments with 3-6 mice per group. Error bars represent ± SEM. **p ≤ 0.01, ***p ≤ 0.001. ns, non-significant.

IL-33 is maintained in the nucleus of epithelial cells under homeostatic conditions, but is released following tissue damage (2). To determine if c-Rel was required for ILC2 function under inflammatory conditions, we used a model of allergic lung inflammation. We treated control WT and c-Rel^-/-^ mice intranasally with the protease allergen papain on day 0, 1 and 2, and examined the acute, ILC2-dependent inflammatory response in the lungs on day 3 (32, 34, 35). Strikingly, c-Rel^-/-^ mice showed reduced papain-induced lung inflammation, with a significantly diminished influx of lymphocytes into BAL and significantly fewer eosinophils (**Figures 4A–C**). Like homeostatic conditions in the BM, we did not find any significant difference in the frequencies and numbers of lung ILC2s in c-Rel^-/-^ and control mice in response to papain (**Figures 4D, E**). However, we observed significantly reduced *Il5* and *Il13* expression in the absence of c-Rel, suggesting that c-Rel plays an important part in the expression of these type 2 inflammatory proteins. (**Figures 4F, G**). As damaged epithelial cells also produce IL-25 and IL-33 in response to injury, which could in turn activate ILC2s in a paracrine manner (2) to produce IL-5 and IL-13, we examined their involvement in this model. Importantly, we found that expression levels of *Il25 and Il33* were equivalent between c-Rel^-/-^ mice and control mice (**Figures 4H, I**), showing that c-Rel does not regulate *Il25* and *Il33* expression in epithelial cells in our experiments. Collectively, these data suggest that c-Rel is an important regulator of ILC2-dependent lung inflammation.

### c-Rel Is Indispensable for IL-33-Dependent Lung Inflammation and Activation

**Figure 5:**
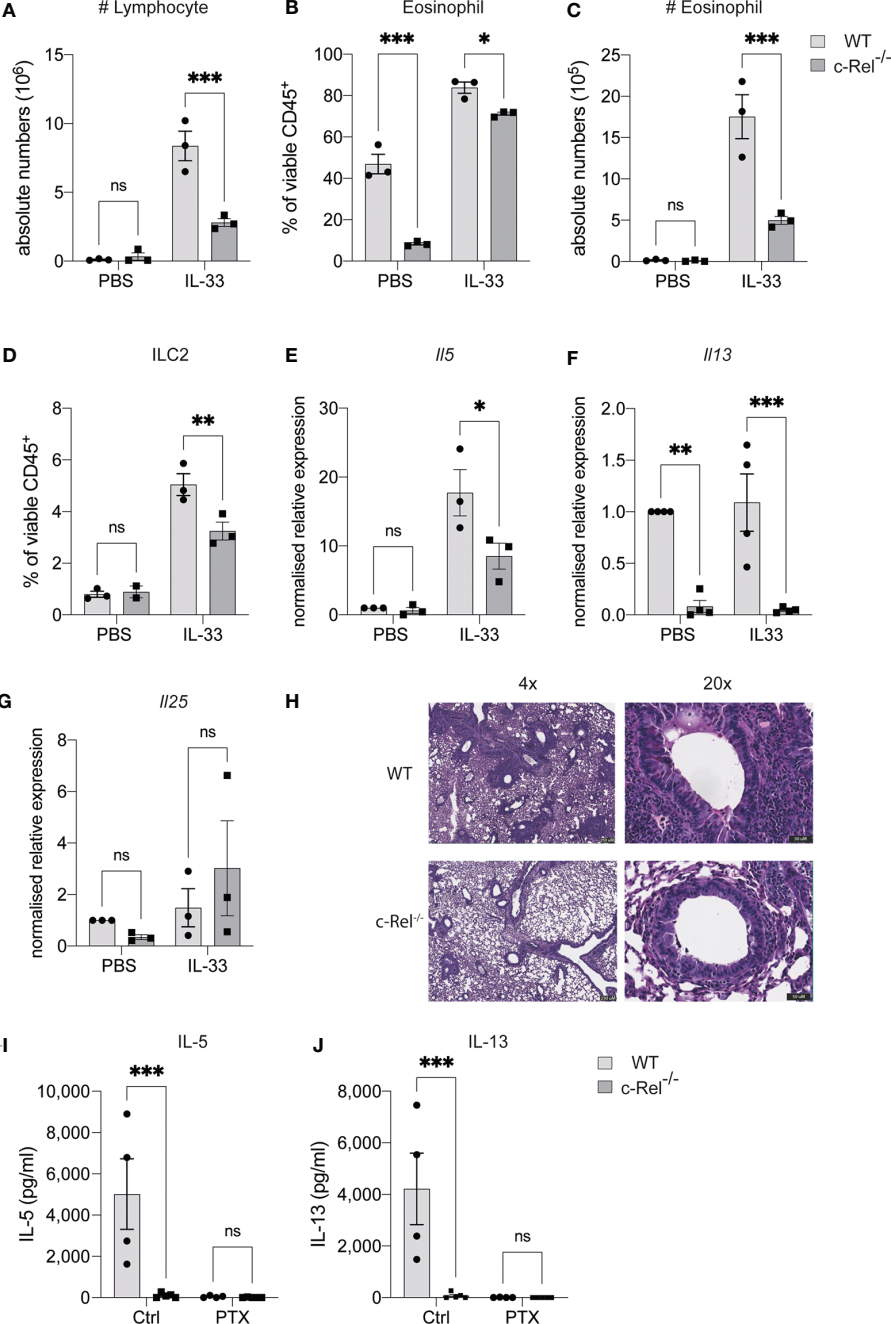
c-Rel is critical for ILC2-mediated lung inflammation, induced by IL-33. **(A)** Absolute numbers of CD45^+^ lymphocytes in the bronchoalveolar lavage (BAL) fluid following PBS (control) or rIL-33 administration for three days. **(B)** Quantification of Siglec-F^+^ CD11c^-^ eosinophils in BAL among viable CD45^+^ cells. **(C)** Absolute numbers of BAL eosinophils. **(D)** Quantification of CD127^+^ KLRG-1^+^ lung ILC2s (gated on Lin^-^ CD90^+^ CD25^+^ ST2^+^) among viable CD45^+^ cells. **(E–G)** qPCR analysis of the indicated genes from lung tissue following PBS or rIL-33 administration. **(H)** PAS-stained lung histology visualized using 4x (left) and 20x (right) magnification. **(I, J)** ELISA analysis of the indicated cytokine from the supernatant of *ex vivo* lung ILC2s cultures in the presence of IL-2, IL-7, IL-25 and IL-33, with or without a c-Rel inhibitor, pentoxifylline. All graphs are representative of at least two independent experiments with 3-5 per group. Error bars represent ± SEM. *p ≤ 0.05, **p ≤ 0.01, ***p ≤ 0.001. ns, non-significant.

As papain treatment induces expression of the ILC2-inducing cytokine IL-33, and given that IL-33 treatment of ILC2s leads to c-Rel activation, we next tested whether c-Rel was required for the direct activation of ILC2s by IL-33 *in vivo*. We treated control WT and c-Rel^-/^ *^-^* mice intranasally with rIL-33 on day 0, 1 and 2, and analyzed lung responses on day 3. Consistent with our lung inflammation model using papain, c-Rel^-/-^ mice displayed reduced IL-33-dependent inflammation in the lungs, with reduced infiltration of lymphocytes and eosinophils into the BAL (**Figures 5A–C**), reduced frequencies of ILC2s in the lungs (**Figure 5D**), and lower expression levels of *Il5* and *Il13*, but not *Il25*, in lung tissue (**Figures 5E–G**). IL-33-induced goblet cell hyperplasia and mucus production in the lungs lacking c-Rel was also severely reduced (**Figure 5H**). Further, *ex vivo* activation of lung-derived ILC2s with IL-2, IL-7, IL-25 and IL-33, resulted in the c-Rel-dependent production of IL-5 and IL-13 (**Figures 5I, J**). Additionally, in the presence of pentoxifylline, an NF-κB inhibitor with selectivity for c-Rel over other family members (36, 37), c-Rel-sufficient ILC2s failed to produce IL-5 and IL-13, in a similar manner to c-Rel-deficient ILC2s. Together, these observations strongly suggest that c-Rel is critical for IL-33-dependent activation of ILC2s during lung inflammation.

## Discussion

Although IL-33 has been shown to be a potent activator of ILC2s during inflammatory responses, the molecular mechanisms responsible for its regulation are poorly understood. While the primary source of IL-33 is epithelial cells (2), other cell types such as natural killer T cells (NKT) cells and alveolar macrophages (38) have also been shown to produce IL-33. Previous studies have established a role for several factors for a number of transcription factors such as GFI1 (39), ETS1 (40), TCF1 (41, 42), G9a (32), and non-canonical NF-κB factors (22, 43) as important regulators of ILC2 biology; however, the roles of canonical NF-κB proteins have not been directly examined in ILC2s. The present study identified the presence of canonical NF-κB family members p50, RelA and c-Rel activity in ILC2s following IL-33 stimulation. We found that while c-Rel is dispensable for early ILC2 precursor development in BM, it is required for optimal ILC2P expansion and proliferation. Furthermore, our results suggest that c-Rel regulates ILC2 activation and function in response to papain- or rIL-33-induced lung inflammation. Overall, the present study identifies the transcription factor c-Rel as a regulator of IL-33-dependent ILC2 function, particularly during lung inflammatory responses.

We observed an increase in nuclear localization and DNA binding of each canonical NF-κB family member p50, RelA and c-Rel in ILC2s upon IL-33 stimulation. Typically, NF-κB can act as a homodimer (e.g., c-Rel/c-Rel), and/or a heterodimer (e.g. c-Rel/p50) (2, 9). Thus, further characterization of the functional roles in different combinations of NF-κB family members in IL-33-stimulated ILC2s is needed. In response to IL-33, the expression of canonical NF-κB in our murine activated ILC2s is consistent with previous findings for human IL-1β-primed ILC2s that require IKK-mediated activation of NF-κB (44). In addition to canonical NF-κB signals, non-canonical NIK-dependent NF-κB signaling involving p100 and RelB activation occurs in pulmonary ILC2s, specifically in response to alveolar macrophage-derived TNF-α *via* TNFR2 (43). Notably, the expression of the canonical NF-κB genes in TNF-α-stimulated ILC2s is either unchanged or reduced (43). Thus, we speculate that the activation of canonical NF-κB signals in pulmonary ILC2s is largely dependent on the pathway downstream of IL-33 mediated by c-Rel, whereas the non-canonical signals are activated in response to other activating cytokines such as TNF-α.

Unlike conventional cytokines, IL-33 is typically released by epithelial cells following damage to barrier tissue and binds to ST2 that forms a heterodimer with IL-1RAcP (45, 46) to serve as an alarmin in response to inflammation and infection (47–49). During the steady state, IL-33 is constitutively expressed in both human (50) and mouse airway epithelial cells (51). Our results show that in steady-state conditions, the absence of either c-Rel or p50 subunit of NF-κB1 does not impair the proportion of ILC2s expressing ST2. However, we observed that the homeostatic development of pulmonary naive ILC2s is somewhat compromised when c-Rel is absent. This indicates that c-Rel-dependent immature ILC2s in the lungs develop independently of ST2 expression. Alternatively, c-Rel may be a prerequisite for IL-33-mediated egress of ILC2s from BM to the lungs (3), which can potentially explain why the accumulation of naive lung ILC2s is slightly reduced in the absence of c-Rel. Notably, there is only a minimal reduction in ILC2s in the lungs of naive mice lacking c-Rel, suggesting that c-Rel is not absolutely required for normal naive pulmonary ILC2 development. This is in line with our earlier observation, in which minimal NF-κB activity is detected in non-activated ILC2s in the absence of IL-33, but its activation is increased upon IL-33 stimulation. Such an observation is consistent with prior studies that have demonstrated the expression and nuclear activity of NF-κB in resting, naive T cells is minimal (52–54).

Numerous studies have demonstrated that both human (55–57) and mouse ILC2s are strongly associated with respiratory and allergic diseases, including asthma and that activation of ILC2s requires IL-33 signals (58, 59). Expression of *Il33* or *Il1rl1* genes also correlates with susceptibility to asthma (19, 60–62), as well as the type 2 response in murine experimental asthma models (2, 58, 59), further supporting a crucial role of IL-33-mediated ILC2s activation during allergy. In addition, blockade of IL-33 signaling has been shown to limit the development of ILC2-mediated chronic asthma (63), with the administration of IL-33 activating ILC2-dependent lung inflammatory responses and goblet cell hyperplasia (12). As the loss of c-Rel impairs *ex vivo* IL-33-dependent ILC2 proliferation and expansion, we questioned whether c-Rel could also be responsible for ILC2-mediated allergic lung responses. Both papain and IL-33 have been shown to induce ILC2-dependent IL-5 and IL-13 production and airway eosinophilia (32, 34, 35). We found that c-Rel is critical for the development of papain- and IL-33-induced allergic lung inflammation. We observed a significantly reduced airway eosinophilic response and lower levels of *Il5* and *Il13* expression in the lungs of c-Rel-deficient mice. Surprisingly, following papain but not IL-33 treatment, the frequencies of lung ILC2s lacking c-Rel remain at the equivalent levels seen in control mice, implying that c-Rel is critically required for ILC2 activation in producing IL-5 and IL-13, but not ILC2 development or survival in response to papain. Collectively, we highlight that c-Rel is important for ILC2-dependent lung inflammation in response to papain and IL-33, with this finding confirming and extending previously published work on the role of c-Rel in promoting ovalbumin-alum induced airway inflammation (64).

c-Rel is responsible for many important immune cell functions controlled by regulating the expressions of a wide range of genes (65), including *Il13* (66) and is therefore also likely to be implicated in IL-13-producing Th2-mediated responses associated with asthma and allergy. Further, the loss of p50 alone, or in combination with the absence of c-Rel is associated with impaired effector CD4 T cell survival (67) and Th2 cell functions following ovalbumin challenge (13), respectively. p50-deficient mice failed to mount lung allergic responses due to reduced GATA3 expression in Th2 cells and impaired type 2 cytokine secretion (13). Given p50 can dimerize with c-Rel (65), this points to a potential role of c-Rel in Th2 cell functions, including the possible involvement of impaired c-Rel dependent Th2 cells functions as part of the explanation for the defective inflammatory responses observed in our c-Rel-deficient mice. However, a comparative analysis of IL-5 and IL-13 production in murine experimental asthma showed that major producers of these type 2 cytokines are ILC2s, rather than Th2 cells (5). Furthermore, in line with our data showing that in cultures of *ex vivo* c-Rel-deficient mice ILC2s or pulmonary WT ILC2s treated with the c-Rel inhibitor, pentoxifylline, both sets of cultured cells failed to produce IL-5 and IL-13. This makes it very likely that the response observed in our *in vivo* asthma model experiments is largely mediated by c-Rel-dependent ILC2s.

NF-κB is not only expressed by immune cells (68), but also non-immune cells such as epithelial cells and stromal cells (69). It has been previously shown that transgenic mice expressing active IκB kinase (IKK) β in airway epithelial cells develop allergic airway disease due to activation of NF-κB signaling that in turn elevates Th2 and ILC2 responses during lung inflammation (70). Conversely, selectively preventing IKK-dependent NF-κB activation in mouse intestinal epithelial cells impairs Th2 responses following helminth infection, resulting in susceptibility (71). Thus, these findings point to an important role of epithelial cell-intrinsic NF-κB signals in mediating the type 2 response. Since we used global c-Rel knockout mice in the present study, we acknowledge that the immune response generated in our lung inflammation models may not be entirely ILC2-intrinsic if the c-Rel-mediated epithelial cell activation is contributing to the response observed. However, we observed equivalent expression of the epithelial cell-derived cytokines *Il25 and Il33* in papain-induced inflamed lungs of control and c-Rel^-/-^ mice, indicating that c-Rel deletion in airway epithelial cells does not affect upstream activation component of the inflammatory response.

Asthma and allergic diseases are rapidly increasing worldwide, highlighting a crucial need for a novel anti-inflammatory drug that can efficiently modulate the type 2 response, known to be central to immune responses responsible for lung inflammation. In addition to Th2 cells, ILC2s are key producers of IL-5 and IL-13. Unlike Th2 cells, ILC2s mainly reside at the interface between the host and environment, such as in the submucosa of lungs, initiating inflammation in response to allergens. Accordingly, the localization of ILC2s provides a strategic approach to specifically target ILC2s to attenuate airway hyperreactivity associated with asthma. Our results suggest that a c-Rel-specific inhibitor such as the IT-603 and IT-901 compounds (72, 73) may offer a novel therapeutic strategy to inhibit IL-33-induced ILC2 activity in diseases such as asthma and allergies.

## Data Availability Statement

The raw data supporting the conclusions of this article will be made available by the authors, without undue reservation.

## Ethics Statement

The animal study was reviewed and approved by Monash University Animal Ethics Committee.

## Author Contributions

Designed the study and conceptualization: CZ, SS, and AZ. Performed the experiments: AZ, SS, JR, GR, and JN. Analyzed and interpreted experimental data: AZ, and SS. Performed the EMSA experiment: RG. Provided mouse strains: TSF, and SG. Wrote and drafted the manuscript: AZ. Provided comments, edited and reviewed the manuscript: CZ, SS, SG and AZ. All authors contributed to the article and approved the submitted version.

## Funding

This work was supported by NHMRC project grants (APP1104433 and APP1104466 to CZ) and Monash University Biomedicine Discovery Scholarship (to AZ).

## Conflict of Interest

The authors declare that the research was conducted in the absence of any commercial or financial relationships that could be construed as a potential conflict of interest.

The handling editor declared a shared affiliation with the authors at the time of review.

## Supplementary Material

Supplementary Figure 1
**(A)** Gating strategies of BM ILC2s (cells, single cells, live cells, CD45^+^ Lin^-^ α_4_β_7_^+^). αLPs were defined as c-KIT^low^ CD25^-^, ChILPs were defined as c-KIT^high^ CD25^-^ and ILC2Ps were defined as c-KIT^low^ CD25^+^. **(B)** Representative histogram plots of GATA expression in BM-derived Lin^-^ α_4_β_7_^+^ ILC2s (gated on CD45^+^ Lin^-^). **(C)** Quantification of GATA3 expression in BM-derived ILC2s. Error bars represent ± SEM. BM, bone marrow; αLP, alpha lymphoid progenitor; ChILP, common helper innate lymphoid progenitors; ILC2Ps, innate lymphoid cell 2 progenitor; ns, non-significant.

Supplementary Figure 2
**(A)** Representative flow cytometry plots of cultured BM-derived ILC2s of WT and c-Rel^-/-^ mice. FACS-sorted BM ILC2Ps were cultured in ILC media in the presence of IL-2, IL-7 and IL-25 for 14 days. On d14, expanded cells were assessed for ILC2 markers (CD45^+^ Lin^-^ CD25^+^ SCA-1^+^). The top plot shows CD45^+^ Lin^-^ ILC2s (gated on live cell), whilst the bottom plot shows CD25^+^ SCA-1^+^ cells (gated on parent CD45^+^ Lin^-^ cells).
