# Supplementary figures and images for: c-Rel Is Required for IL-33-Dependent Activation of ILC2s
Source: Front Immunol. 2021 Jun 14;12:667922. doi: 10.3389/fimmu.2021.667922 (PMC8236704; doi:10.3389/fimmu.2021.667922)

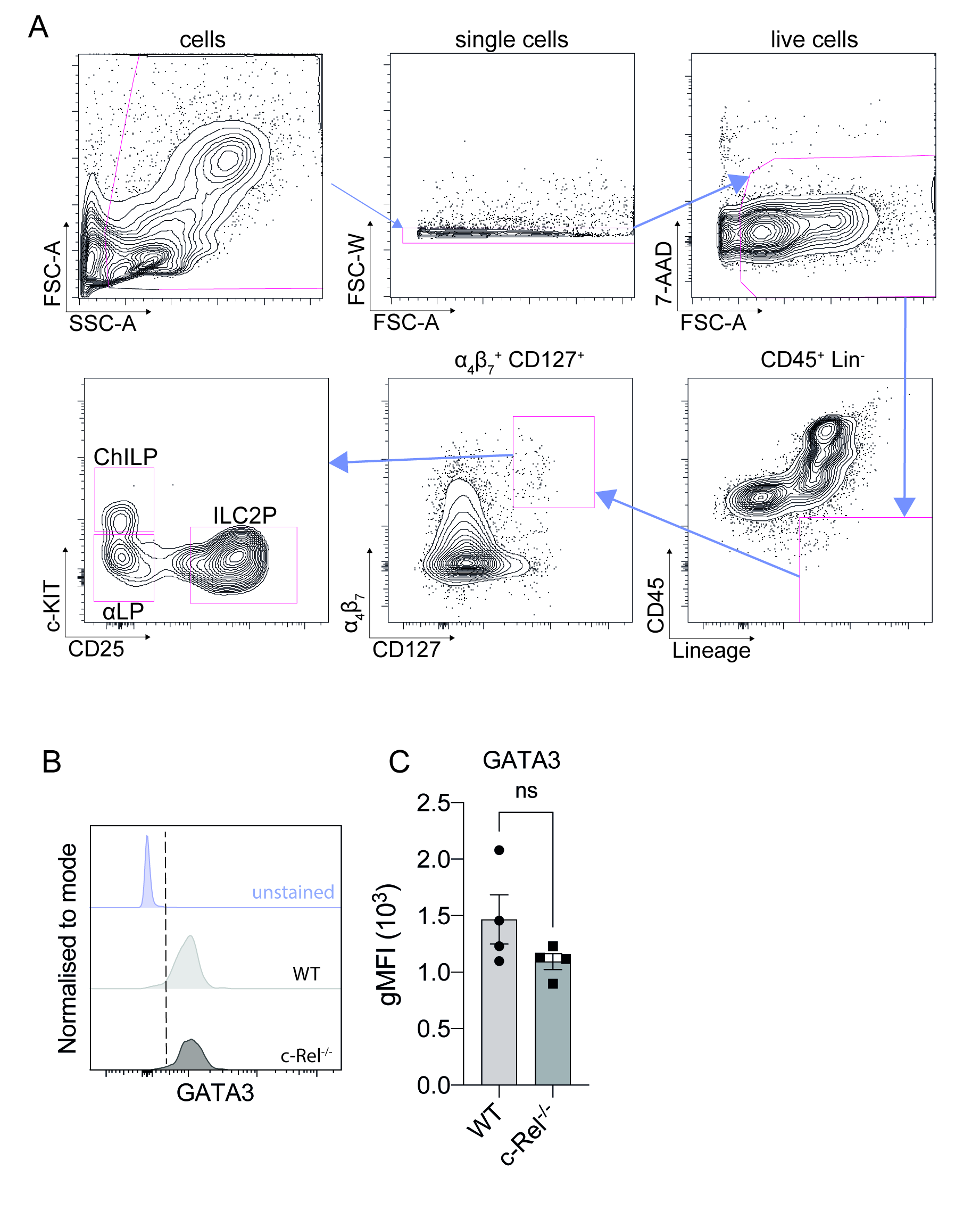

Supplement: Supplementary Figure 1 — (A) Gating strategies of BM ILC2s (cells, single cells, live cells, CD45+ Lin- α4β7 +). αLPs were defined as c-KITlow CD25-, ChILPs were defined as c-KIThigh CD25- and ILC2Ps were defined as c-KITlow CD25+. (B) Representative histogram plots of GATA expression in BM-derived Lin- α4β7 + ILC2s (gated on CD45+ Lin-). (C) Quantification of GATA3 expression in BM-derived ILC2s. Error bars represent ± SEM. BM, bone marrow; αLP, alpha lymphoid progenitor; ChILP, common helper innate lymphoid progenitors; ILC2Ps, innate lymphoid cell 2 progenitor; ns, non-significant. [file Image_1.tif]

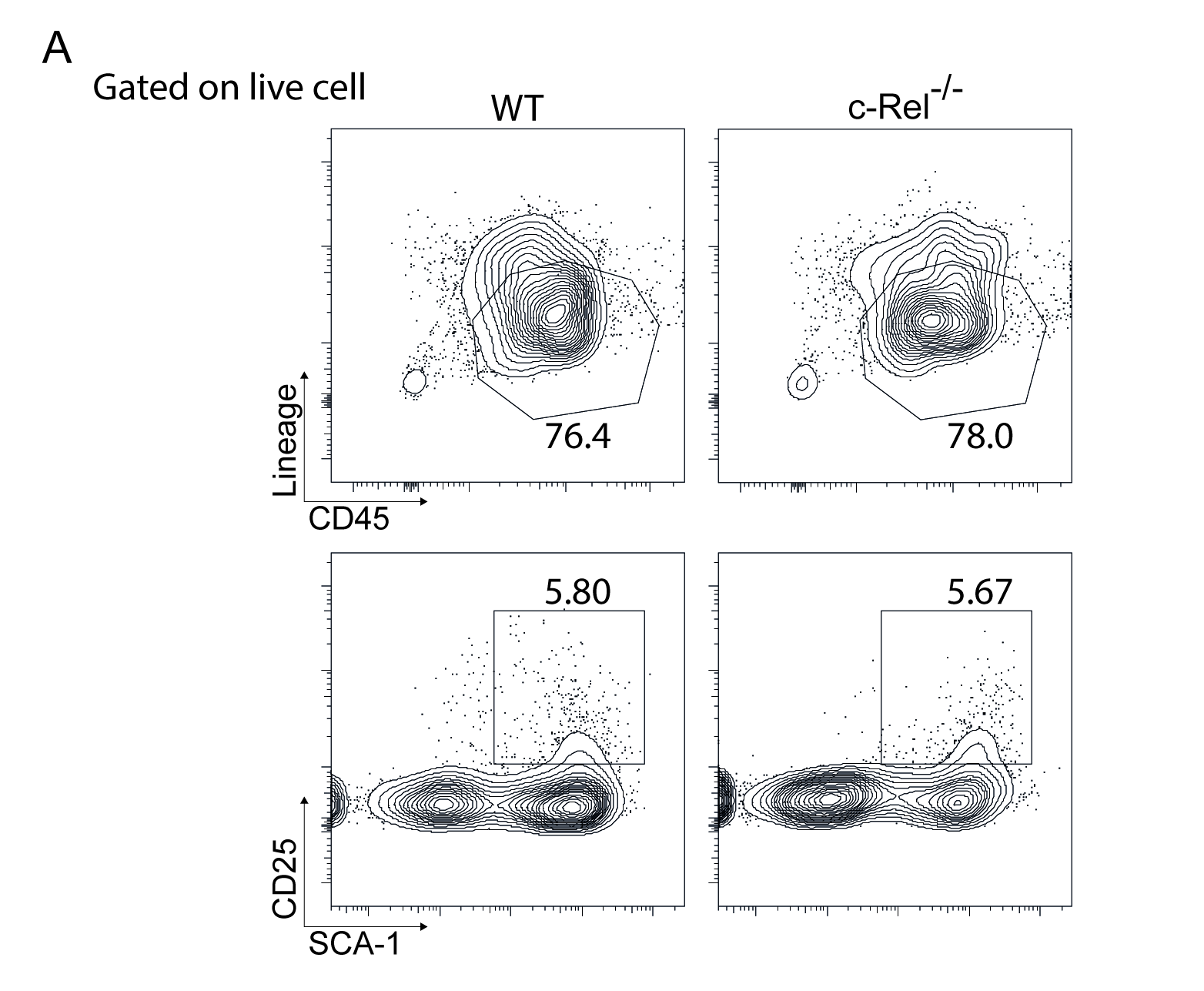

Supplement: Supplementary Figure 2 — (A) Representative flow cytometry plots of cultured BM-derived ILC2s of WT and c-Rel-/- mice. FACS-sorted BM ILC2Ps were cultured in ILC media in the presence of IL-2, IL-7 and IL-25 for 14 days. On d14, expanded cells were assessed for ILC2 markers (CD45+ Lin- CD25+ SCA-1+). The top plot shows CD45+ Lin- ILC2s (gated on live cell), whilst the bottom plot shows CD25+ SCA-1+ cells (gated on parent CD45+ Lin- cells). [file Image_2.tif]
