# Supplementary material for: c-Rel Is Required for IL-33-Dependent Activation of ILC2s
Source: Front Immunol. 2021 Jun 14;12:667922. doi: 10.3389/fimmu.2021.667922 (PMC8236704; doi:10.3389/fimmu.2021.667922)
Supplement: Supplementary file 3 [file Table_1.pdf]

Supplementary Table 1. Flow cytometry antibodies

Antibody	Clone	Supplier
CD3ε	145-2C11	Tonbo Biosciences
CD4	GK1.5	Tonbo Biosciences
CD5	53-7.3	Tonbo Biosciences
CD8	53-6.7	Tonbo Biosciences
CD11b	M1/170	Tonbo Biosciences
CD11c	N418	eBioscience
CD19	eBio1D3	eBioscience
CD45R (B220)	RA3-B62	eBioscience
MHCII	M5/114	eBioscience
Ly6G/C	RB6-8C5	eBioscience
Ter119	TER-119	Tonbo Biosciences
NK1.1	PK136	eBioscience
$\alpha_4\beta_7$	DATK32	eBioscience
Sca-1	D7	eBioscience
CD25	P661.5	eBioscience
c-KIT (CD117)	2B8	eBioscience
CD127	SB/119	BD Bioscience
CD45	30-F11	eBioscience
ST2 (IL-33R)	RMST2-2	eBioscience
KLRG1	2F1	eBioscience
CD25	P661.5	eBioscience
CD90.2	53-2.1	eBioscience
CD127	SB/119	BD Bioscience
CD45	30-F11	eBioscience
Siglec-F	E50-2240	BD Bioscience
GATA3	TWAJ	eBioscience
