# Supplementary material for: c-Rel Is Required for IL-33-Dependent Activation of ILC2s
Source: Front Immunol. 2021 Jun 14;12:667922. doi: 10.3389/fimmu.2021.667922 (PMC8236704; doi:10.3389/fimmu.2021.667922)
Supplement: Supplementary file 4 [file Table_2.pdf]

Supplementary Table 2. qPCR primers

Gene of interest	Forward primer (5'-3')	Reverse primer (5'-3')
Actb	ACTAATGGCAACGAGCGGTTC	GGATGCCACAGGATTCCATACC
Il5	GATGAGGCTTCCTGTCCCTACTC	TCGCCACACTTCTCTTTTTGG
Il13	CCTGGCTCTTGCTTGCCTT	GGTCTTGTGTGATGTTGCTCA
Il25	ACAGGGACTTGAATCGGGTC	TGGTAAAGTGGGACGGAGTTG
Il33	TCCAACTCCAAGATTTCCCCG	CATGCAGTAGACATGGCAGAA
cRel	TGCGACCTCAATGTGGTGAG	TATTTGGGGCACGGTTGTCA
